# Wearable biosensor systems and resilience: a perfect storm in health care?

**DOI:** 10.3389/fpsyg.2014.00853

**Published:** 2014-08-06

**Authors:** Robert L. Drury

**Affiliations:** ReThink Health, Bainbridge Island, WA/University of Wisconsin Institutes for DiscoveryMadison, WI, USA

**Keywords:** resilience, heart rate variability, psychobiological health marker, complex adaptive systems, risk vs. resilience factors, consilience, digital epidemiology

## Abstract

We begin by placing our discussion in the context of the chronic crisis in medical care, noting key features, including economic, safety and conceptual challenges. Then we review the most promising elements of a broadened conceptual approach to health and wellbeing, which include an expanded role for psychological, social, cultural, spiritual and environmental variables. The contributions of positive and evolutionary psychology, complex adaptive systems theory, genomics and neuroscience are described and the rapidly developing synthetic field of resilience as a catalytic unifying development is traced in some detail, including analysis of the rapidly growing empirical literature on resilience and its constituents, particularly heart rate variability. Finally, a review of the use of miniaturized ambulatory data collection, analysis and self-management and health management systems points out an exemplar, the Extensive Care System (ECS), which takes advantage of the continuing advances in biosensor technology, computing power, networking dynamics and social media to facilitate not only personalized health and wellbeing, but higher quality evidence-based preventive, treatment and epidemiological outcomes. This development will challenge the acute care episode model typified by the ER or ICU stay and replace it with an ECS capable of facilitating not only healthy autonomic functioning, but both ipsative/individual and normative/population health.

The laudable goal of this special issue is to further elucidate the role of autonomic dysregulation in important conditions including anxiety disorders such as PTSD. We endeavor to present an integrated view of a particular combination of conceptual, methodological and technological resources which are related to these important issues and, at the same time, reach beyond it, to overarching scientific and social issues. This broad perspective is warranted, I believe, because we, as a national society and even world confluence of cultures are experiencing a crisis regarding the provision of effective, affordable and safe health care and support of well-being. During a recent invited presentation to the Chinese Academy of Science's Institute of Psychology, I was reminded that the ancient Chinese pictograph for crisis combines danger and opportunity. A pointed example of this is the situation in the United States where we spend huge amounts of money to provide services which frequently have very limited and sometimes dangerous outcomes. Not only is this danger tangible to millions of patients but the fiscal danger to our country is significant. If health care costs continue to escalate at the present rate, our Federal government may be bankrupt by 2040 (Longman, 2013)

Financial catastrophe is only one danger related to the current system, most often described as a procedure and profit driven disease treatment approach. Patients routinely face dangers of both improper and inadequate treatment of iatrogenic disease (Institute of Medicine, 1999, 2006). While these problems persist in the face of polarized political ineptitude and massive vested interests which are frequently inimical to both safety and efficacy, the major obstacle is adherence by most health care professionals, including their suppliers and vendors of medical supplies and equipment to the tenants of the infectious disease biomedical model (Drury, 2013; Emmaneul, 2014). While Emmanuel's proposals are the basis of the Affordable Care Act, and include adoption of the electronic medical record, use of social media and other personal communication technologies, changing reimbursement for providers from procedure to outcome based and stringent quality of outcome measures, they don't include the scientific elements to be described here. These scientific constituents represent a major opportunity to contribute to human health and well-being.

We are now on the cusp of a progressive transformation which some have identified as revolutionary or true Kuhnian paradigm succession, based on evolutionary and systems science approaches in biology, psychology, neuroscience and other disciplines. Originating in mathematics, the study of complex adaptive systems has gradually spread to biology and then the psychosocial sciences. This continuing development places the person in a hierarchy of nested systems from the subatomic to the cosmological (Strogatz, 1994; Johnson, 2001; Mitchell, 2009). Rather than sole focus on pathology and aggressive treatment, this approach gives recognition to the factors that promote human resilience and positive adaptive outcomes. In the health area this has been described a personalized medicine, prescriptive medicine and 4P medicine, well-articulated by Hood (2014) of the Institute for Systems Biology. It has proven a generally integrative force, although the process of radical scientific discovery and subsequent translation into practice has frequently progressed in Max Planck phrase “one funeral at a time.” Eric Topol (2012) has advocated the “creative destruction of medicine” to indicate the degree of transformation needed. These developments have even led one of our most distinguished scientists, Wilson (1998) to proclaim the goal of consilience, which is the comprehensive unification of knowledge. The rapid development of omics also turns toward systematization of broad areas of inquiry from genomics to connectomics [the Human Connectome Project (National Institutes of Health, 2012)]. Our understanding and ability to intervene to promote health and well-being will be greatly enhanced by these conceptual developments in science with psychology functioning as a central hub science, since our understanding of human life and human nature is the primary lens through which we view all other phenomena.

This special issue focuses on an area of huge conceptual and empirical significance, since our understanding of human nature from a complex systems perspective emphasizes the complicated interactive dynamics of the many constituent systems that comprise the person and their sociocultural environment. In particular the role of the autonomic nervous system is increasingly understood as a major contributor to systems regulation and intermodulation of multiple systems beyond the central nervous system, including the cardiovascular, digestive, respiratory and immune systems. The major significance of Porges' polyvagal theory (2011) and Thayer et al.'s (2009) neurovisceral integration model is illustrated not only by their frequent citation, but the invited presence of their work in this issue.

Both Porges and Thayer have emphasized the important role played by the vagal nerve complex in bidirectional mediation of CNS-cardiac interaction from a neurobiological perspective. Porges use of the term polyvagal highlights the several functions of the vagal nerve complex, especially in facilitating the social engagement system. Thayer et al. (2012) suggests that heart rate variability (HRV) is an excellent indicator of vagal regulatory activity in the service of ongoing dynamic neurovisceral integration and concludes his recent meta-analysis of HRV (2012) proposing that HRV may function as “a proxy for ‘vertical integration’ of the brain mechanisms that guide flexible control over behavior with peripheral physiology, and as such provides an important window into understanding stress and health.” A recent Frontiers of Psychology article by Park and Thayer (2014) describes how cardiac vagal tone modulates perceptual and attentional processes in response to emotional stimuli and he comments on implications of HRV for health and well-being. Following the work of both Porges and Thayer, the role of the vagal nerve is not confined to only CNS-cardiac interaction, since it is anatomically connected with a variety of other important systems such as the immune, respiratory and digestive systems and the facial expression of emotion. Thus, HRV seems to be implicated in a wide variety of important relationships regarding health maintenance and disease processes. It is a sensitive indicator of both the presence of a wide variety of disease and illness conditions and health and well-being, and has been used as a measure for assessing the effects of treatment interventions as well. Some of the conditions using HRV recently published include sepsis, sudden cardiac death, diabetes, insomnia and sleep apnea, cerebral palsy, infection post coronary artery bypass surgery, dysauatonomia, effects of air pollution, need for air evacuation in triage and syncope.

This rapidly growing line of research is contributing empirically and theoretically to the overall neuroscientific literature on the role of structure and dynamics of the central nervous system and its complex regulatory interactions with other bodily systems. This area was recently reviewed by Pessoa (in press) focusing on brain networks, rather than individual regions. In addition to HRV studies, some important emerging areas will be cited. The study of the adaptive function of the brain's default network (Andrews-Hanna, 2012) is giving a more nuanced understanding of previously “dark areas” of the CNS. Basar's (2013) studies of brain oscillations emphasize the importance of multimethod studies to most adequately understand the function of the brain, and his comments apply as well to this chronobiological approach to the interaction of all biological systems. The work of Gotlib's group (Hamilton et al., 2013) highlights the neural systems approach to psychopathology, but this approach is equally relevant and germane to other elements of illness. The recent work of Sandman's group (Pincus et al., 2014) ties the use of systems and network conceptualization to the emergence of resilience, emphasizing the use of non-linear dynamics as a previously neglected tool to elucidate biological functions that do not meet the requirements or assumptions of traditional linear methods inherited from classical physics. The work of Calhoun's group (Stephen et al., 2013) identifies independent component analysis as a viable contributor to the study of such complex phenomena.

The two major elements of the complex systems analysis approach advocated here are resilience and HRV. Resilience is a conceptual development that integrates the many critiques of the tradition pathology-oriented medical treatment system. Zautra and his colleagues have described resilience as the vehicle for the emergence of a new model of health (Reich et al., 2012). Zautra's group and other prominent scientists (Lukey and Tepe, 2008; Southwick and Charney, 2012; Spira and Drury, 2012) and well informed popular writers such as Andrew Zolli (2012) have presented well supported descriptions of resilience with a transdisciplinary emphasis. Resilience is generally defined as the ability of an individual, group, organization or culture to “bounce back” adaptively after experiencing a challenge, stressor or trauma and maintain its mission, purpose or goal structure. Rather than identify risk factors for adverse outcomes, it uses the massive data accumulated by the sciences to explore resilience factors which promote positive adaptational outcomes such as growth development and optimal functioning, as summarized in the citations above. Important methodological and conceptual elements such as extensive longitudinal data collection, multimethod studies and appropriate statistical approaches have been emphasized (Reich et al., 2012). Other elements of central scientific importance are the roles of emotion regulation (Gross, 2007; Kring and Sloan, 2010) and stress and allostasis (Drury et al., 2010; Karatsoreos and McEwen, 2013).

Some of the key resilience factors that have been identified (Drury, 2013) are cognitive coping and appraisal, realistic optimism, social support, religion and spirituality and sense of meaning and purpose in life, and psychophysiological self-soothing and affect regulation. It should be noted that resilience is not strictly an individual characteristic, but also applies to groups, communities, and cultures. Morality, defined by Greene (2014) as a “set of psychological adaptations to allow otherwise selfish individuals to reap the benefits of cooperation,” is a resilience characteristic at various levels. The work on compassion, empathy and altruism by the Stanford Center for the Study of Compassion and Altruism Research and Education (CCARE, 2014) is contributing to our understanding of resilience, as does the work of Peterson and Seligman (2004) on character strengths, virtues and positive psychology. As exemplified by Kabat-Zinn (2013), Mindfulness-Based practices have been associated with positive resilience outcomes as have practices that develop Emotional Intelligence (Goleman, 2005). Further research is needed to refine our understanding of interrelationships between various resilience factors.

One of those resilience factors is HRV, a rapidly growing field of inquiry which has led to over 16,000 peer reviewed references in a recent Pub Med search. While disturbance of HRV is associated with a huge variety of disorders and conditions, increased HRV is associated with higher levels of health, well-being and optimal performance. HRV refers to the variation in interbeat interval which is characteristic of the heart, with diminished HRV most often representing impaired function, and greater HRV showing greater health and functional ability. Notably, HRV disturbance is not only an indicator of physiological and biomedical functioning, but also reveals the effects of psychosocial disturbances and conditions. The majority of articles assembled by Dr. Ginsberg in this special issue use HRV as a central variable in studying both autonomic function and dysfunction, especially as it relates to important problems such as PTSD and mTBI. HRV is both an element in the treatment of PTSD (Gewirtz and Lehrer, this issue), but is also a highly sensitive marker of psychobiosocial health status which can be applied in diagnosis/treatment and health promotion, disease prevention and performance optimization.

Resilience and HRV are poised to be key components in the nascent Personalized Health and 4P Health movements, which are premised on an entirely different approach to cultivating and supporting human assets, including health and well-being. Not only does it sometimes take multiple episodes of “scientific” discovery to break paradigmatic logjams, which are by nature conservative, but as in Plank's quotation above, there is sometimes active resistance to new and innovative approaches.

The traditional model has focused on massively expensive and “heroic” episodes of disease treatment, epitomized by the urgent visit to the ER and Intensive Care Unit. The emerging integral approach (Rakel, 2012; Drury, 2013) suggests the following components: ongoing frequent, or even continuous monitoring of health status, using unobtrusive microelectronic data acquisition and analysis devices, networked with cloud-based high throughput analytic algorithms to track ongoing psychobiosocial functioning. The 4P model described by Hood (2014) highlights personalized, predictive, prescriptive and participatory systems of care, with each individual involved in self-regulation activities closely related to their current health status, including both assets and challenges. This approach can be mediated through the use of smart phones, social media, sophisticated non-linear algorithms and other rapidly growing technologies that synergize this important field. As mentioned earlier, the use of a evolutionarily-grounded complex adaptive systems overall framework will obviate many of the structural impediments to the transition from traditional to integral health and wellbeing.

This unfolding development is based on a biobehavioral understanding of human nature informed by current evolutionary science. From that perspective, the various challenges thrust upon contemporary individuals can be seen as dramatically different from those of the last two million years of hominid experience. A telling example of this issue is the challenge of modern treatment to “cure the disease epidemic of obesity.” While some treatments claim temporary benefit, most often scientific study has revealed yo-yo patterns with little long term weight loss. The long term evolutionary success of the savanna based gorge and starve model which has been historically conserved is strongly implicated in the limited success of conventional approaches and suggests again, that we must shift our understanding and be historically acute and informed about our nature and the limits and limitations on adaptation.

Another salient aspect of the emerging model is its recognition of the central role in human and other mammalian life of attachment phenomena (Bowlby, 1988). Long ignored by psychology and other relevant disciplines, attachment was dismissed as too ethereal and fuzzy to be studied scientifically. Both Bowlby and Harlow fired the opening shots across the bow of organized science and we are now in a very generative phase of exploring the development and problem issues surrounding attachment issues. The recent volume by Gillath et al. (2012) has reinforced the importance of the conceptual and methodological issues described here in their focus on relationship science. They specifically emphasize the role of neuroscience, evolutionary biology and socio-cultural studies to gain a more complete understanding of human relationships. Examples are the contribution of neurobiology in the area of neuroplasticity (McEwen and Gianaros, 2011) and neurochemistry in the area of oxytocin (Carter, 2014), both of which stimulate much current research relevant to human health and its upper limits.

The perfect storm alluded to in my title, occurs when a set of individual factors produce a seemingly unexpected and potentially very disruptive synthesis. If you happen to be in a fishing boat in the middle of such an event, or a patient in a modern medical care system, the situation can become more that disruptive and actually dangerous, even life-threatening. The synergy involves not only many classes of environmental factors, but individual behaviors as well. I will sketch only one example of the type of process/outcome that offers an alternative to the limitations of traditional medicine. The maintenance of the traditional system has been sustained for only the briefest historical moment from an evolutionary standpoint despite the tremendous costs, financial and human.

An urgent symptom leading to an ambulance transport to an ER and then ICU is the archetype of dramatic and aggressive health care mediated by high technology. Its high priority in the attentional focus of many consumers is shown by the great popularity of the TV show with that title. Here I will suggest an alternative that may not have the same appeal to viewers of reality TV, but will use technology in a more nuanced and potentially large scale manner creating an Extensive Care System (ECS). An exemplar of a functional ECS would include a longitudinal data acquisition and analysis system for a population sample based on unobtrusive microelectronic hardware to obtain continuous HRV data which functions as a highly sensitive marker of psychobiological status. Using the rapidly growing abilities of high throughput and cloud based computing, mediated through smart phones, this data could be used algorithmically to not only scan for potential impairment but also encourage increasingly greater levels of wellness. The use of advanced data analyses, including fractal and other non-linear approaches is increasingly recognized as optimal for detecting some organic life function and dysfunction that is not detectable apparent standard statistical methods, especially the puny methods that are sometimes paraded as “gold standard” clinical trials. The use of sophistication technology with innovative analytic methodology may give us much more effective interventions for both treatment and prevention, as Vodopivec-Jamsek et al. (2012) have proposed in their Cochrane Database Systems Review of mHealth. An ECS approach is an ideal vehicle to increase our use and understanding of self-control and its evolution (MacLean et al., 2014) in encouraging patient participation in the maintenance of health and well-being.

Following the tradition of learning theory based psychology, such a system would offer positive reinforcement, social or tangible, for objective indications of positive health behavior as indicated in either accelerometer-based activity level or high level of HRV. Consistent with our understanding of behavior change and maintenance, immediate reinforcement is the most effective approach to encouraging health behaviors. A second category of impaired health status information would also be used to prompt an individual to engage in adaptive preventive and health promoting activities, which could also be subsequently reinforced. Of note many health insurers, companies and local governments are exploring methods to reinforce positive health behaviors, although the lack of an agreed upon metric is problematic. As pointed out above, weight loss, even when reinforced by reduced health premiums or other reinforcers, may be ineffective and/or lead to gaming the system. Beyond prompting of individual, the ECS is capable of notifying designated health professionals of decreases in health status with decision support for triage and intervention options. Of note, King et al. (2009) has recently conducted research using brief HRV data analysis generated at crash sites to improve decision support efficiency of triaging Life Flight evacuations, potentially saving both lives and money.

This ECS approach borrows from health psychology and epidemiology the necessity of longitudinally repeated measures of the same individual to create an ipsative/normative data base very sensitive to potential disruption common in complex adaptive systems, which are notoriously “sensitive to initial conditions.” While needing to protect personal health information, this approach would collect literally millions of data points from which firm baselines of healthy functioning could be obtained, possibly for the first time ever. The acceptability and popularity of such an approach is suggested by the millions of individuals who voluntarily participate in the “Quantified Self” and other social media movements, which like other forms of self and alternative care, are funded directly by the participant with no government or business based subsidy. In addition to the values of longitudinal data collection, use of small world network dynamics and analytics, transdisciplinary study and electronic health data acquisition and archiving, this approach is easily tasked to study populations in pursuit of public health and epidemiology goals. Especially in poor regions where smart phones are leapfrogging older communication strategies, this example of the ECS could massively impact the attainment of both prompt intervention through Digital Epidemiology (Wolfe, 2011) and assessing and supporting higher levels of positive health status with little traditional infrastructure. In particular, this example avoids some of the pitfalls of approaches that rely on self-report or low fidelity molar measures such as weight or BMI.

All the components of the proposed ECS currently exist, awaiting a more complete integrative dialectical synthesis. Similar to climate change, it is not clear how difficult and disastrous the consequences of maintaining the current system through inaction will be. Highlighting the central functions and dysfunctions of the autonomic nervous system addressed in this issue, the ECS will be difficult to operationalize and systematically deploy, dependent on zeitgeist readiness but offers a relatively distinct way forward. Of course this path forward needs focused resources to accomplish such a translational research and development objective but the time may be right for the self-organizing emergence of existing conceptual, empirical and technological resources in a prosocial Perfect Storm.

## Conflict of interest statement

The author declares that the research was conducted in the absence of any commercial or financial relationships that could be construed as a potential conflict of interest.
